# Sleep Problems and Quality of Life in Breast Cancer Patients

**DOI:** 10.3390/curroncol32090508

**Published:** 2025-09-12

**Authors:** Andreas Hinz, Michael Friedrich, Thomas Schulte, Mareike Ernst, Ana N. Tibubos, Katja Petrowski, Nadja Dornhöfer

**Affiliations:** 1 Department of Medical Psychology and Medical Sociology, University of Leipzig, 04103 Leipzig, Germany; 2 Rehabilitation Clinic Bad Oexen, 32549 Bad Oeynhausen, Germany; 3 Department of Clinical Psychology, Psychotherapy, and Psychoanalysis, University of Klagenfurt, 9020 Klagenfurt, Austria; 4 Department of Nursing Science, Diagnostics in Healthcare & eHealth, University of Trier, 54286 Trier, Germany; 5 Department of Psychosomatic Medicine and Psychotherapy, University Medical Center, Johannes Gutenberg University of Mainz, 55131 Mainz, Germany; 6 Department of Medical Psychology and Medical Sociology, University Medical Center, Johannes Gutenberg University of Mainz, 55131 Mainz, Germany; 7 Department of Gynecology, University Hospital Leipzig, 04103 Leipzig, Germany

**Keywords:** sleep problems, sleep quality, insomnia, quality of life, breast cancer

## Abstract

Background: Sleep problems are frequently observed in breast cancer patients. However, the relationship between sleep quality and overall quality of life (QoL) and the specificity of different sleep-related questionnaires have not yet been adequately studied in breast cancer patients. Methods: The sample of this cross-sectional study consisted of 533 breast cancer patients, recruited in a German rehabilitation clinic, with a mean age of 52.3 years (SD = 12.5 years). The following three sleep-related questionnaires were used: the Pittsburgh Sleep Quality Index (PSQI), the Insomnia Severity Index (ISI), and the Jenkins Sleep Scale (JSS). In addition, we used the QoL instrument EORTC QLQ-C30. Results: Sleep quality was poor in this sample of breast cancer patients. The effect sizes *d*, indicating the difference in sleep quality between the patient sample and the general population, were between 0.97 and 1.76 (*p* < 0.001). QoL was impaired in all components (*p* < 0.001); the impairment in the dimension of sleep quality (*d* = 1.70) was among the highest. Sleep quality was correlated with all components of QoL. The comparison of the three sleep-related questionnaires showed that the results obtained in oncological studies partly depend on the instrument used. Conclusion: As the burden of sleep problems is high, screening for sleep problems in breast cancer patients is important.

## Simple Summary

It is well known that breast cancer patients often suffer from sleep problems. These problems have a negative impact on patients’ quality of life. In research studies and in routine clinical practice, often only a single instrument is used to measure sleep quality. It therefore remains unclear to what extent the study results can be generalized. In the present study, several instruments were used in parallel to record sleep quality. This allows conclusions to be drawn about the generalizability of the results and about the dependence of sleep quality on various clinical and socio-demographic factors. This provides starting points for a targeted and effective assessment of sleep problems and their treatment.

## 1. Introduction

Breast cancer is the most commonly diagnosed cancer in women worldwide, with an estimated 2.3 million new cases diagnosed in 2020, accounting for around 25% of all new cancer cases in women [1]. While the worldwide incidence of breast cancer is increasing, largely due to epidemiological and demographic transitions, as well as the extensive use of mammography screening [2], the breast cancer mortality rates are decreasing in many European countries [3]. In Germany, approximately 70,000 women are diagnosed with breast cancer each year [4]. After the introduction of mammography screening between 2005 and 2009, the rate of newly diagnosed cases initially rose and then fell slightly. Since 2000, breast cancer mortality rates have been declining steadily. The relative 10-year survival rate for women in Germany is 83%, and the relative 10-year survival rate is 67% [4].

Sleep problems are a common and significant issue for breast cancer patients [5,6,7,8,9,10]. A systematic review and meta-analysis [11] indicated that breast cancer patients have a mean sleep disturbance prevalence of approximately 60%, which is higher than that observed in patients with other cancer types. This is corroborated by findings from a meta-analysis on breast cancer survivors [12] that reported a mean prevalence of poor sleep quality of 62%. The cumulative evidence suggests that sleep quality is a critical factor that should be monitored and managed in breast cancer care to improve both QoL and survivorship. It has been shown that sleep problems in breast cancer patients are associated with distress, anxiety and depression [13,14,15], pessimism [16], pain and fatigue [5,13], socioeconomic factors such as income levels [17], side effects of cancer treatments such as chemotherapy or hormone therapy [18], treatment adherence [19], and even poorer survival rates [20,21].

Sleep problems can be placed in the context of quality of life (QoL). It is well-known that QoL is significantly impaired in breast cancer patients [22,23,24,25]. However, the significance of sleep problems in the context of overall QoL in breast cancer patients has not yet been sufficiently investigated. There remain questions, such as to what extent sleep quality is more or less impaired in comparison to other components of QoL, and to what extent sleep problems correlate with restrictions in other areas of QoL. These concerns beg the question of whether sleep problems are associated more with the physical or the psychological components of QoL.

Regarding age differences in sleep quality among breast cancer patients, the results are inconsistent. While some researchers found higher rates of sleep problems in the older age range [5,26], other studies [27] failed to detect such differences. Moreover, the question of whether the associations between sleep problems and general QoL are age-dependent has not yet been investigated.

Since effective treatments for sleep disturbances exist, e.g., cognitive behavioral therapy [28,29] or exercise intervention [30], it is important to precisely identify patients suffering from sleep disturbances.

Various questionnaires have been developed to effectively assess sleep quality. Frequently used instruments are the Pittsburgh Sleep Quality Index (PSQI) [31], the Insomnia Severity Index (ISI) [32], and the Jenkins Sleep Scale (JSS) [33]. In addition, several instruments with a broader scope contain one item representing sleep problems such as the European Organisation for Research and Treatment of Cancer Quality of Life Questionnaire EORTC QLQ-C30 [34], the Patient Health Questionnaire-9 (PHQ-9) [35] for measuring depression or the General Health Questionnaire GHQ-12 [36].

Most empirical studies analyzing sleep quality in cancer patients used only one of these questionnaires. However, as the different questionnaires focus on slightly different content and have different structures, it is difficult to assess the extent to which the results found on the relationship between sleep quality and other factors depend on the specific questionnaire used. For this reason, three different questionnaires for recording sleep quality, sleep problems or insomnia were used in parallel in this study. The correlations between the questionnaires and the systematic comparison of the correlative structures with other variables were intended to provide information on the extent to which the different instruments actually measure the same variable, and thus the extent to which the results obtained with an individual instrument can be generalized.

The objectives of this study were (a) to investigate the prevalence of sleep problems and detriments in QoL in breast cancer patients in comparison with sleep and QoL in women from the general population, (b) to compare three established questionnaires for measuring sleep problems, (c) to analyze the associations between sleep problems and general QoL to determine the role of sleep problems in the context of general physical and mental health problems, and (d) to investigate age effects in sleep problems.

## 2. Materials and Methods

### 2.1. Sample of Cancer Patients

This is a cross-sectional study that followed the Strengthening the Reporting of Observational Studies in Epidemiology (STROBE) guidelines (Supplementary Materials) [37]. Study participants were consecutively recruited in a German oncological rehabilitation clinic between July 2022 and June 2023. In Germany, cancer patients are offered the opportunity to participate in rehabilitation programs to help restore their physical, mental and social functioning, with a typical duration of three weeks. At the beginning of the rehabilitation program, the patients were informed about the study and asked to participate. If the patients agreed, they were given a booklet with the questionnaires which they typically completed within the first two days of the rehabilitation program. After the questionnaires were returned, a study nurse checked them for completeness and, in the event of missing values or ambiguities, asked the patients to fill them out completely. The inclusion criteria for this study were a confirmed cancer diagnosis, age 18 years and above, sufficient command of the German language, and absence of severe cognitive impairment. A total of 2250 patients with any cancer diagnosis were eligible and therefore asked to participate in the study, and 1733 (77%) of them agreed to take part. In our analyses, we limit ourselves to women with breast cancer (*n* = 533). Written informed consent was obtained from all participants. The study was approved by the Ethics Committee of the Medical Faculty of the University of Leipzig, approval number: 513/21-ek.

### 2.2. Instruments

For assessing sleep problems, we used three questionnaires, the PSQI, the ISI, and the JSS.

– **PSQI.** The Pittsburgh Sleep Quality Index (PSQI) [31] consists of 19 items that can be assigned to the following seven components of sleep quality: subjective sleep quality, sleep latency, sleep duration, sleep efficiency, sleep disturbances, use of sleep medication, and daytime dysfunction. Four of the items consist of time specifications, while the remaining items have a four-level response pattern. In addition to the seven subscales (range 0–3), a global score of overall sleep quality can be calculated by adding up the scores of these seven dimensions. This results in a sum score ranging from 0 to 21, with a high value indicating a high level of sleep problems. Sum scores above 5 are generally considered as indicating poor sleep. Normative values of the PSQI, derived from a large German general population sample, are available [38].
– **ISI.** The Insomnia Severity Index (ISI) [32] is based on the diagnostic criteria for insomnia outlined in the Diagnostic and Statistical Manual of Mental Disorders (DSM-IV) and the International Classification of Sleep Disorders (ICSD). The questionnaire consists of seven items on insomnia symptoms and their impact on daily functioning, which cover the following components: sleep onset, sleep maintenance, early morning awakening, satisfaction level with current sleep pattern, interference with daily living, noticeability of impairment due to the sleep difficulty, and level of distress caused by the sleep problem. For each of the items there are five response options, coded as 0–4. This results in a sum score range from 0 to 28. The ISI sum scores can be assigned to categories as follows: no significant insomnia (0–7), subthreshold insomnia (8–14), moderate insomnia (15–21), and severe insomnia (22–28) [32].
– **JSS.** The Jenkins Sleep Scale (JSS) [33] is an instrument for measuring sleep problems with four items: trouble falling asleep, waking up several times per night, trouble staying asleep, and waking up tired. For each item, there are six response options, ranging from 0 to 5, which results in a sum score range from 0 to 20. There is no generally accepted cutoff for poor sleep quality, though proposals for cutoffs have been made, e.g., ≥12 for sleep problems [39], or 0–9 (none/some), 10–14 (moderate), 15–20 (severe sleep problems) [40]. Normative values of the JSS, derived from a large German general population sample, are available [41].

In addition to the sleep-related questionnaires, the following instruments were used:

– **EORTC QLQ-C30.** The EORTC QLQ-C30 [34] was developed for measuring QoL in cancer patients. The instrument consists of 30 items that are assigned to five functioning scales, a global health status/QoL scale, three symptom scales, and six single-item scales. One of these single-items scales refers to sleep problems with the question “Have you had trouble sleeping?”. Each scale of the EORTC QLQ-C30 is transformed to the range from 0 to 100, with higher functioning scores and lower symptom scores representing better QoL. Normative values of the EORTC QLQ-C30 are available [42,43]. A sum score of the EORTC QLQ-C30 can be calculated aggregating the functioning scales and the (inverted) symptom scales [44].
– **GAD-7.** The Generalized Anxiety Disorder Screener GAD-7 [45] is a screening instrument designed to detect symptoms of generalized anxiety disorder according to the DSM-IV. The item scores range from 0 to 3, resulting in a sum score range from 0 to 21.
– **PHQ-9.** The Patient Health Questionnaire-9 [35] is a screening instrument with nine items, designed to assess depression. For each item., the patients are asked to assess how much they were bothered by the symptoms over the last two weeks. For each item, there are four response options (0–3), resulting in a sum score range from 0 to 27.

In the event of missing values in the questionnaires, the missing values were replaced by the mean of the valid items of the scale, provided that at least half of the items were valid.

### 2.3. Statistical Analysis

Effect sizes *d* were calculated to indicate group mean differences. These coefficients relate the difference between the group means to the pooled standard deviation. The association between the sleep scales as well as the associations between the sleep scales and the other QoL scales were expressed in terms of Pearson correlations. The reliability (internal consistency) of the sleep scales was determined with Cronbach’s α coefficient.

Age differences were statistically tested with analyses of variance (ANOVA) with three age groups: 18–40 years, 41–60 years, and ≥61 years.

To compare the patients’ scores with those of the general population, we used normative data taken from large representative general population studies. Regarding the EORTC QLQ-C30, we used a publication on European mean scores [43], which presents aggregated data for six European studies. Because of the similarity with the German normative scores, we preferred to use the larger “European” sample with the following strategy: We used the women’s mean scores and standard deviations of the age decades as reported in the normative table. The age decades were weighted according to the frequencies of the age decades in the breast cancer sample. For example, the percentage of patients in the age group 40–49 years was 27.1% in this study. These percentages were taken as the weighting factors for calculating the general population mean value of the scales of the EORTC QLQ-C30. This approach means that the breast cancer sample and the female general population sample are nearly identical in terms of age distribution.

Regarding the GAD-7 and the PHQ-9, this procedure was not possible since the publications of the general population normative scores for the GAD-7 [45] and the PHQ-9 [46] did not present the results in terms of suitable age decades. Therefore, we used the mean scores of the total female general population reported in these normative studies.

The relationships between sleep problems and components of quality of life were initially expressed using Pearson correlations. In addition, multiple regression analyses were calculated, in which the components of QoL were considered as dependent variables, and all sociodemographic and clinical variables listed in Table 1 were used as independent variables in addition to the respective sleep scale. All calculations were performed with SPSS, version 27.

## 3. Results

### 3.1. Characteristics of the Sample

A total of 2250 cancer patients of all diagnoses were eligible and asked to participate, and 77% of these (*n* = 1733) agreed to take part in the study and to complete the questionnaire. Of these, there were 554 female and 6 male patients with breast cancer. Only one female cancer patient had to be excluded due to missing values in accordance with the criteria specified in the methods section. In our study, we restrict the analyses to the 553 female breast cancer patients with complete data. Their mean age was 52.3 years (SD = 12.5 years). Most of them were employed and received surgery, and frequent treatments were radiation therapy, chemotherapy, and hormone therapy. Further characteristics are listed in Table 1.

### 3.2. Comparisons Between Breast Cancer Patients and the General Population

Table 2 presents the patients’ mean scores of three sleep-related questionnaires, the QoL questionnaire EORTC QLQ-C30, and two other questionnaires measuring aspects of mental health. In those cases where normative scores were available, these scores are also presented in Table 2. The effect sizes indicate the amount of the mean score difference between the groups.

Breast cancer patients slept markedly worse than women of the general population, the effect sizes of the group differences were between 0.97 and 1.76. Since there are no normative data for the ISI, no effect size could be calculated for this instrument.

Using the generally accepted threshold for poor sleep for the PSQI (scores above 5), 434 patients (78.5%) were poor sleepers. The four categories of the ISI resulted in the following percentages: no significant insomnia (18.3%), subthreshold insomnia (30.4%), moderate insomnia (36.5%) and severe insomnia (14.8%). Using the cutoff value of 12 in the JSS, this resulted in a proportion of 62.0% for poor sleep.

Regarding QoL, the cancer patients showed lower mean scores in all functioning scales and higher scores in all symptom scales in comparison with groups from the general population, indicating worse QoL in the group of cancer patients. Out of the symptom scales, fatigue (*d* = 1.76) and insomnia (*d* = 1.70) presented the strongest effect sizes. Regarding mental health, the difference in depression (*d* = 1.21) was slightly stronger than that for anxiety (*d* = 0.94). With one exception (constipation), the mean differences for all scales of the EORTC QLQ-C30 were greater than 10 points and thus clinically relevant according to the frequently used criterion for clinically relevant mean differences [47].

### 3.3. Associations Between the Sleep Quality Instruments PSQI, ISI, and JSS

The correlations between the three sleep instruments were as follows: *r*(PSQI, ISI) = 0.79, *r*(PSQI, JSS) = 0.71, and *r*(ISI, JSS) = 0.86. The internal consistency coefficients (Cronbach’s α) were as follows: α (PSQI) = 0.79, α (ISI) = 0.90, and α (JSS) = 0.83.

### 3.4. Relationship Between Sleep Problems and QoL

Table 3 shows that all functioning scales correlated negatively, and all symptom scales correlated positively with the three sleep instruments. In addition to these correlations, standardized regression coefficients beta are given that express the strength of the relationship between sleep problems and components of QoL considering sociodemographic and clinical covariables. Since the impact of the covariables was low, the beta coefficients are similar to the correlation coefficients.

The highest correlations between the sleep instruments and the dimensions of the EORTC QLQ-C30 were found for the one-item scale insomnia (*r* between 0.68 and 0.77). Out of the remaining scales, fatigue was most strongly correlated with sleep problems. The comparison between the scales physical functioning and emotional functioning showed that their respective associations with sleep problems were of a similar magnitude.

The comparisons between the three instruments with regard to their correlations with the other scales showed generally higher correlations for the PSQI and the ISI in comparison with the JSS. Depression (PHQ-9) was more strongly correlated with sleep problems than anxiety (GAD-7) in all three sleep instruments.

### 3.5. Age Differences in Sleep Problems and Associations Between Sleep Problems and QoL

Table 4 illustrates age differences captured by each sleep scale. The highest levels of sleep problems were found for the middle age group (41–60 years) in all three questionnaires, while the youngest and the oldest groups were less affected by sleep problems. The ANOVA results were as follows: PSQI: *F* = 2.18, *p* = 0.115; ISI: *F* = 5.34, *p* = 0.005; JSS: *F* = 6.49; *p* = 0.002.

Regarding the correlations between sleep problems and general QoL in terms of the EORTC QLQ-C30 sum score, Table 4 repeats the findings of Table 2 in that the associations were weakest for the JSS in comparison with the PSQI and the ISI. It further shows that the correlations are strongest for the oldest patients.

## 4. Discussion

The first aim of this study was to examine the frequency of sleep problems in breast cancer patients. The percentage of poor sleepers was 78.5% for the PSQI and 62.0% for the JSS (with the cutoff 12). Regarding the ISI, the percentage was 51.3% for the combined category moderate and severe insomnia, and 81.7% for those who reported at least subthreshold insomnia. This shows that sleep problems are frequent in breast cancer patients and that the precise frequency of poor sleep depends on the questionnaire and the cutoff used. Norm values were available for two of the three questionnaires, and the patients’ mean scores were markedly above those of the general population: *d* = 0.97 (PSQI) and *d* = 1.76 (JSS). The effect size obtained from the 1-item sleep scale of the EORTC QLQ-C30 was also very high (*d* = 1.70). The absolute mean score difference between the two groups, breast cancer patients and the general population, in the insomnia scale was 38.6 points, which is markedly higher than the often used threshold for clinically meaningful differences of 10 points [47]. This finding is remarkable insofar as sleep problems are also common in the general population. Standardization studies in various countries report the following prevalence rates of poor sleep quality, using the PSQI: Austria: 32% [48], Germany: 36% [38], and Hong Kong: 39% [49]. The differences between the effect sizes obtained with the different instruments in our study might be due to the different contents of the questionnaires. While the JSS and the sleep scale of the EORTC QLQ-C30 focus on the subjective evaluation of sleep quality, the PSQI also includes more objective components such as sleep duration. Although all three sleep questionnaires (PSQI, ISI, and JSS) are validated instruments for assessing sleep quality, they sometimes produce different results. The proportion of patients with good sleep according to the JSS (38.0%) is almost twice as high as the proportion according to the PSQI (21.5%). For clinical practice and research, this means that if only one instrument is used to assess sleep quality, as is usually the case, the results are likely to be somewhat inaccurate. At least when it is necessary to assess sleep quality as accurately as possible, the use of two different instruments should be considered.

QoL was also markedly impaired in the breast cancer sample, all scales showed worse QoL scores with effect sizes between 0.39 and 1.79. The symptom scales with the highest differences in terms of effect sizes were fatigue (*d* = 1.76) and sleep problems (*d* = 1.70); these effect sizes were stronger than those obtained for other symptoms such as pain (*d* = 0.77). While the importance of fatigue has been documented in multiple studies [50,51], the relevance of sleep does not seem to be as evident for many clinicians. Sleep disturbances can have significant negative impacts on patients’ physical and mental well-being, highlighting the importance of addressing this issue as part of comprehensive cancer care.

The associations between sleep quality and the other aspects of QoL were investigated by establishing the correlations between the three sleep scales and the components of the EORTC QLQ-C30. Unsurprisingly, the highest correlations were found for the EORTC QLQ-C30 scale insomnia since it is closely linked with sleep problems. Out of the remaining components of QoL, the highest associations were found for fatigue with correlations between 0.42 and 0.53. This underlines the strong relationship between sleep and fatigue. In this context it is important to note that these correlations cannot be used to derive causal relationships. Sleep problems can cause fatigue, fatigue can cause sleep problems, and finally it is conceivable that both, sleep problems and fatigue, are influenced by a third factor. Nevertheless, it is interesting to note that sleep problems are associated with all functioning scales to a relatively similar magnitude. Therefore, neither the physical component of QoL nor the mental component of QoL is particularly strongly or weakly associated with sleep quality.

Numerous studies have shown that sleep problems, fatigue, and depression are strongly correlated. This means that these three symptoms can occur in clusters. However, if all variables are collected via self-assessment (as in the present study), a certain response bias or acquiescence response style [52,53] may contribute to this correlation, meaning that the correlations would be lower if objective methods were used to assess sleep quality.

In terms of correlations with anxiety and depression, all three sleep scales showed slightly higher associations with depression (*r* between 0.57 and 0.67) compared to anxiety (*r* between 0.48 and 0.57). However, it must be taken into account here that the PHQ-9 already contains an item on sleep quality, which partly contributes to the correlations with the sleep scales.

There was no linear age dependency of the sleep problems. On all three sleep scales, the values were highest for the middle age group (41–60 years) and lower in the other two age groups (18–40 years and above 60 years), and these age differences were statistically significant for two of the three questionnaires. This non-linear trend may also explain the inconsistent findings evident in the literature with regard to age dependency [5,26,27]. If the analyses are restricted to the comparison of only two age groups, such a non-linear age dependency does not become apparent.

The correlations between sleep quality and general QoL were similar for all age groups with only slightly stronger correlation coefficients in the oldest age group. This means that sleep quality in the context of overall QoL has approximately the same significance for all age groups.

There are effective treatment methods for disturbed sleep, e.g., medication, relaxation therapy [54], exercise interventions [55,56], or cognitive-behavioral therapy [57]. It is therefore important to identify patients who can benefit from these interventions. The questionnaires developed for assessing sleep quality are suitable tools in principle, but our study has shown that the various established questionnaires do not lead to exactly the same results. A special methodological feature of this study is the parallel use of three established questionnaires to record sleep quality. The correlations between these questionnaires ranged from 0.71 to 0.86, with the strongest being between the ISI and the JSS. This means that each of the instruments has its specific features in addition to their shared focus on sleep quality. As mentioned above, the PSQI differed most from the other instruments. It explicitly measures seven different components of sleep, whereas the ISI and JSS items do not have such a subscale structure. In addition to the subjective overall view of sleep quality, which is expressed by subscale 1 of the PSQI, this instrument also includes other, more objective components of sleep, such as sleep duration. However, despite its significantly larger number of items, the correlations between the PSQI and the other QoL scales were not higher than those of the one-dimensional sleep scales. For a global assessment of subjective sleep quality, a shorter questionnaire such as the ISI or the JSS would therefore appear to be sufficient. In principle, however, the PSQI offers the advantage of separately analyzing different components of sleep quality could be analyzed separately.

Several limitations of this study should be mentioned. All of the sleep instruments used here reflect the subjective view of sleep and not the objective sleep parameters that can be recorded using polysomnography methods. Studies on the relationship between objective and subjective sleep parameters show relatively low correlations between these recording approaches [58,59,60]. It therefore remains unclear to what extent the results found here would have been repeated if objective methods had been used. The associations found in this study can possibly be attributed, at least in part, to certain response tendencies in the sense of social desirability. The sample of breast cancer patients was restricted to those treated in a rehabilitation clinic. Patients experiencing lower levels of distress may not make use of such a rehabilitation treatment, and patients with too much distress may not be able to participate in this rehabilitation procedure. Thus, our sample probably contains a disproportionate number of patients with a medium level of distress. Furthermore, we did not analyze the specific components of sleep problems, which in principle would have been possible with the PSQI. Finally, due to the cross-sectional design of the study, no causal statements can be made about the relationship between sleep problems and QoL.

## 5. Conclusions

The study confirmed the magnitude of sleep problems in breast cancer patients and the relevance of sleep problems in the context of QoL. The comparison of the three questionnaires showed that the results on sleep quality partly depend on the instrument used, which must be taken into account when interpreting the relevant studies.

## Figures and Tables

**Table 1:** Sociodemographic and clinical characteristics of the sample (*n* = 553).

Characteristics	*N*	%
Age group		
18–40 years	110	19.9
41–60 years	291	52.6
≥61 years	152	27.5
Education ^(a)^		
Elementary school (8–9 years)	75	13.6
Junior high school (10 years)	198	35.9
High school/university (≥11 years)	271	50.4
No formal qualification	1	0.2
Employment status ^(a)^		
Employed	380	69.0
Unemployed	26	4.7
Retired	121	22.0
Other	24	4.4
Time since diagnosis		
≤12 months	321	58.0
>12 months	232	42.0
Treatment		
Surgery ^(a)^		
No	5	0.9
Yes	548	99.1
Chemotherapy ^(a)^		
No	201	36.5
Yes	350	63.5
Radio therapy ^(a)^		
No	72	13.0
Yes	481	87.0
Hormone therapy ^(a)^		
No	175	31.9
Yes	374	68.1
Antibody therapy ^(a)^		
No	413	75.8
Yes	132	24.2

^(a)^ Missing data not reported.

**Table 2:** Comparison between breast cancer patients and general population samples regarding sleep quality, QoL, and mental health.

Scales	Breast Cancer		General Population		*d*	*p*
	M	(SD)	M	(SD)		
Sleep scales						
PSQI	9.1	(4.2)	5.4	(3.4)	0.97	***
ISI	14.3	(6.7)	-	-	-	
JSS	12.7	(5.2)	4.2	(4.7)	1.76	***
Quality of life (EORTC QLQ-C30)						
Functioning scales						
Physical	74.6	(19.9)	92.0	(13.6)	−1.04	***
Role	56.0	(29.3)	90.1	(19.3)	−1.40	***
Emotional	50.7	(28.4)	82.7	(19.0)	−1.35	***
Cognitive	53.3	(30.4)	93.4	(14.3)	−1.79	***
Social	56.8	(29.1)	93.2	(16.8)	−1.59	***
Global QoL	60.7	(19.5)	74.2	(17.6)	−0.73	***
Symptom scales						
Fatigue	54.6	(27.0)	16.4	(16.4)	1.76	***
Nausea/Vomiting	7.3	(17.1)	2.2	(9.2)	0.39	***
Pain	38.0	(30.9)	17.2	(23.1)	0.77	***
Dyspnea	38.1	(31.8)	7.1	(17.8)	1.25	***
Insomnia	62.2	(33.6)	13.6	(23.6)	1.70	***
Appetite loss	15.8	(26.8)	4.1	(13.3)	0.58	***
Constipation	15.7	(27.6)	3.2	(12.1)	0.63	***
Diarrhea	14.9	(26.2)	2.8	(12.4)	0.63	***
Financial difficulties	31.7	(33.3)	4.9	(16.3)	1.08	***
Sum score	65.0	(17.9)	91.7	(14.9)	−1.63	***
Mental health						
GAD-7 (Anxiety)	7.2	(5.0)	3.2	(3.5)	0.94	***
PHQ-9 (Depression)	8.3	(5.1)	3.1	(3.5)	1.21	***

M: mean; SD: standard deviation; *d*: effect size of the group difference; ***: *p* < 0.001.

**Table 3:** Correlations between sleep problems and QoL.

Scales	PSQI		ISI		JSS	
	*r*	Beta	*r*	Beta	*r*	Beta
EORTC QLQ-C30						
Functioning scales						
Physical	−0.47	−0.46	−0.46	−0.46	−0.38	−0.40
Role	−0.42	−0.41	−0.41	−0.41	−0.30	−0.32
Emotional	−0.44	−0.46	−0.49	−0.49	−0.40	−0.41
Cognitive	−0.43	−0.44	−0.50	−0.50	−0.44	−0.43
Social	−0.36	−0.37	−0.41	−0.42	−0.33	−0.33
Global QoL	−0.47	−0.49	−0.48	−0.49	−0.38	−0.38
Symptom scales						
Fatigue	0.52	0.51	0.53	0.54	0.42	0.43
Nausea/Vomiting	0.24	0.24	0.19	0.18	0.14	0.14
Pain	0.44	0.43	0.40	0.40	0.33	0.33
Dyspnea	0.32	0.29	0.26	0.26	0.20	0.22
Insomnia	0.71	0.73	0.77	0.78	0.68	0.70
Appetite loss	0.28	0.26	0.25	0.26	0.20	0.22
Constipation	0.28	0.27	0.23	0.23	0.15	0.16
Diarrhea	0.20	0.22	0.17	0.18	0.15	0.16
Financial difficulties	0.30	0.31	0.30	0.29	0.28	0.26
Sum score	−0.62	−0.62	−0.62	−0.62	−0.50	−0.52
Mental health						
GAD-7	0.51	0.53	0.57	0.57	0.48	0.48
PHQ-9	0.57	0.59	0.67	0.67	0.59	0.60

All correlation coefficients *r* and regression coefficients beta are statistically significant with *p* < 0.001.

**Table 4:** Age differences in sleep quality and in correlations between sleep quality and general QoL (EORTC QLQ-C30 sum score).

	Mean Scores and Standard Deviations						Correlations with General QoL		
Age Group	PSQI		ISI		JSS		PSQI	ISI	JSS
	M	(SD)	M	(SD)	M	(SD)	*r*	*r*	*r*
18–40 years	8.5	(4.0)	13.6	(6.6)	12.7	(5.0)	−0.60	−0.59	−0.49
41–60 years	9.4	(4.3)	15.2	(6.8)	13.3	(5.1)	−0.61	−0.61	−0.49
≥61 years	9.0	(4.0)	13.1	(6.6)	11.4	(5.4)	−0.64	−0.65	−0.54

## Data Availability

Data raw data supporting the conclusions of this article will be made available by the corresponding author on reasonable request.

## Supplementary Materials

The following supporting information can be downloaded at: https://www.mdpi.com/article/10.3390/curroncol32090508/s1, STROBE Statement—Checklist of items that should be included in reports of cross-sectional studies.
